# Efficacy of extended focused assessment with sonography for trauma using a portable handheld device for detecting hemothorax in a low resource setting; a multicenter longitudinal study

**DOI:** 10.1186/s12880-022-00942-y

**Published:** 2022-12-01

**Authors:** Stephen Mbae Kithinji, Herman Lule, Moses Acan, Lauben Kyomukama, Joshua Muhumuza, Patrick Kyamanywa

**Affiliations:** 1 Faculty of Clinical Medicine and Dentistry, Department of Surgery, Kampala International University Western Campus, PO. Box 70, Ishaka-Bushenyi, Uganda; 2 Injury Epidemiology and Prevention Research Group, Division of Clinical Neuroscience, University of Turku, Turku, Finland; 3 Department of Radiology, Mbarara University of Science and Technology, Mbarara, Uganda; 4 Uganda Martyrs University, Nkozi, Uganda

**Keywords:** eFast, Hemothorax, Efficacy, X-ray

## Abstract

**Introduction:**

Chest trauma is one of the most important and commonest injuries that require timely diagnosis, accounting for 25–50% of trauma related deaths globally. Although CT scan is the gold standard for detection of haemothorax, it is only useful in stable patients, and remains unavailable in most hospitals in low income countries. Where available, it is very expensive. Sonography has been reported to have high accuracy and sensitivity in trauma diagnosis but is rarely used in trauma patients in low income settings in part due to lack of the sonography machines and lack of expertise among trauma care providers. Chest X-ray is the most available investigation for chest injuries in low income countries. However it is not often safe to wheel seriously injured, unstable trauma patients to X-ray rooms. This study aimed at determining the efficacy of extended focused assessment with sonography for trauma (eFAST) in detection of haemothorax using thoracostomy findings as surrogate gold standard in a low resource setting.

**Methods:**

This was an observational longitudinal study that enrolled 104 study participants with chest trauma. Informed consent was obtained from all participants. A questionnaire was administered and eFAST, chest X-ray and tube thoracotomy were done as indicated. Data were analysed using SPSS version 22. The sensitivity, specificity, predictive values, accuracy and area under the curve were determined using thoracostomy findings as the gold standard. Ethical approval for the study was obtained from the Research and Ethics Committee of Kampala International University Western Campus REC number KIU-2021-53.

**Results:**

eFAST was found to be superior to chest X-ray with sensitivity of 96.1% versus 45.1% respectively. The accuracy was also higher for eFAST (96.4% versus 49.1%) but the specificity was the same at 100.0%. The area under the curve was higher for eFAST (0.980, *P* = 0.001 versus 0.725, *P* = 0.136). Combining eFAST and X-ray increased both sensitivity and accuracy.

**Conclusion:**

This study revealed that eFAST was more sensitive at detecting haemothorax among chest trauma patients compared to chest X-ray. All patients presenting with chest trauma should have bedside eFAST for diagnosis of haemothorax.

## Background

Focused Assessment with Sonography in Trauma (FAST) is a bedside test developed in the mid 1990’s for use in acute trauma patients to rapidly assess for intra-abdominal hemorrhage and to rule out clinically significant pericardial tamponade [1]. The Extended Focused Assessment with Sonography in Trauma (eFAST) adds additional views of the hemithoraces to look for signs of pneumothorax and haemothorax [2]. These include the right and left pleural spaces (anterior axillary line between 6th and 9th intercostal spaces), and left and right anterior pleural spaces (midclavicular line between 2nd and 3rd intercostal spaces) [2].

The unique features of high frequency resolution ultrasound to differentiate each individual tissue densities, being non-invasive, portable handheld device and non-traumatic makes it preferable to conventional radiography and nuclear medicine [3] especially in unstable trauma patients requiring prompt intervention to save life.

Trauma is one of the leading causes of mortality and disability life years lost worldwide [4]. The trauma burden is highest in low and middle income countries (LMICs) (WHO, 2017). In Africa, trauma is the number one cause of deaths amongst individuals in their productive youthful stage and its resulting mortality is disproportionately higher compared to other regions of the world [5]. Chest trauma is one of the most important and commonest injuries that require timely diagnosis [6], accounting for 25–50% of trauma related mortality globally Eyo et al. [7]. Considerably, East Africa experiences a significant burden of chest injuries. According to Chalya et al. [8] chest trauma contributed to 44% of all injuries due to road traffic accidents seen at Bugando Medical Centre in Tanzania and was responsible for up to 24.2% mortality. In Uganda chest trauma contributed to 34.7% of road traffic injury cases seen at the Country’s national referral hospital in Central Uganda [9] with a resulting mortality rate of 17%. A similar mortality (16.9%) was reported at Mbarara regional referral hospital in Western Uganda [10].

Diagnosis of chest injuries is a challenge in low income countries due to limited access to computed tomographic scan that is deemed the gold standard. Furthermore, the readily accessible chest radiographs are associated with immense costs especially for the multiply injured [11]; exposure to radiation [12] and overcrowding of the emergency department due to waiting lists. Extended focused assessment with Sonography for trauma (eFAST) which can be done at the bedside, has been introduced as a potential diagnostic tool [13]. However, there is limited published data on the accuracy and applicability of this cost-effective and radiation free tool in the detection of traumatic hemothorax in our settings.

Chest X-rays are the most available method of investigating chest injuries in low income countries thus assumed to be the gold standard in this context but the X-ray machines are often malfunctioning, besides pose potential exposure to ionizing radiations enough for cancer development [14]. Ultrasound is cheap, accessible, and fast and can be performed by bed-side without interrupting resuscitation or worsening injuries during transfer. To date, minimal efforts have been made in Uganda to incorporate eFAST in standard operating procedures for investigating haemothorax and haemo-pneumothorax in chest trauma, despite it being cheaper than a chest X-ray and the growing body of evidence for eFAST use for this purpose amidst carrying no risk of radiation exposure [13]. To the best of our knowledge, this was the first study that assessed eFAST in detecting hemothorax comparing it to chest X-ray taking thoracostomy findings as the gold standard in low and middle income countries. This study was aimed at determining the efficacy of eFAST in detection of haemothorax using thoracostomy findings as surrogate gold standard in a low resource setting.

## Study methods

### Study design

This was a two center observational longitudinal study and patients were followed from admission to completion of surgical intervention observing for findings on eFAST, X-ray and surgical intervention.

### Study setting

The study was conducted at the accident and emergency (A&E) and the radiology departments of Kampala International University teaching hospital (KIU-TH) and Mbarara regional referral hospital (MRRH) in south-western Uganda.

### Study population

All patients with traumatic chest injuries who attended the A&E and radiology departments of KIU-TH and MRRH during the one year period from 1st May 2021 to 30th April 2022 were considered for the study.

### Sample size estimation

Daniel’s formula for determining the sample size was used [15]. According to the study on epidemiology of motorcycle injuries presenting to Uganda’s national referral hospital, Mulago; traumatic chest injuries contributed to 34.7% of all trauma injuries [9]. Using the formula N = Z^2^P (1 − P)/d^2^; where N = the sample size, Z = Score corresponding to 95% of confidence interval which is 1.96, P = Proportion of chest injured patients which is 34.7%; (1 − P) = 65.3%, and E = margin error rate set at 5%; the sample size N = 348. To increase the internal validity of study and catering for non-responders, the calculated sample was increased by 10% giving an estimated sample size of 383.

Adjusting sample size to the finite population, Sample size N = ns = (1 + (ns − 1)/n). Where N = adjusted population size, ns = estimated sample size, n = population under study = 142 (based on the hospital data registry), N = 104. Therefore, a sample size of 104 participants with chest injuries was considered for study duration of 12 months.

### Sampling technique

Consecutive recruitment method was used to enroll all eligible participants until the required sample size was realized. 69(66.3% of the sample size) was recruited from MRRH and the remaining 35(33.7%) from KIU-TH using proportionate sampling.

### Eligibility criteria

#### Inclusion criteria

All patients with chest injuries who consented were recruited in the study.

#### Exclusion criteria

Unstable patients that had thoracostomy before chest X-ray, those with massive hemothorax, cardiac tamponade and patients with documented evidence of pleural effusion prior to the trauma event were excluded from the study.

### Training of research assistants

Five surgery residents including the principal investigator participated in the study and were trained in the use of point of care ultrasound. The team was availed with the “point of care ultrasound in resource limited environments” (PURE) model manual [16], which consists of core concepts of emergency ultrasound such as focused assessment for trauma, with aim to establish competence in knowledge related to the indication of the scan, image acquisition, interpretation and integration of findings into patient management. Later the team attended a four weeks’ intensive practicum on use of eFAST in chest and abdominal trauma evaluation and using data collection tools.

The PURE model involves use of the electronic “point of care” ultrasonography (POCUS) manual [17], didactic lectures embedded with videos, followed by practical sessions and knowledge retention assessment test [16]. This training module has been validated in Kenya in similar settings [18] and is accredited by the African Federation for Emergency Medicine [18]. The principal trainers were qualified radiologists from Uganda who were experienced in the use of FAST and eFAST. The training was facilitated by the investigator and trainees. Because of the concerns on learning curve, the investigator and research assistants continued to work under supervision of qualified sonographers and radiologists throughout the study period.

### Data collecting tools

Data for this study was collected using investigator administered checklist. The key variables of interest included demographics, injury mechanisms and patterns, presence or absence of haemothorax, haemo-pneumothorax, nature of surgical intervention and findings on ultrasound, CXR and Tube thoracotomy. The two investigative techniques eFAST and CXR were compared with the findings on tube thoracostomy. The findings at tube thoracostomy were used as the surrogate gold standard to confirm if the investigations correctly detected the haemothorax.

### Data collection procedure

After attending to and excluding the life threatening airway emergencies in the primary survey, the researchers explained the study and its purpose to the participants in order to obtain an informed consent document with a signature or thumb print. However in the event of suspected massive haemothorax or tension pneumothorax, eFAST was performed as part of primary survey and intervention made immediately before the administration of the questionnaire. A pretested check list of parameters of interest was used by the investigator with his data collection team at the radiology and accident and emergency departments. ATLS principles were used in initial assessment and management with eFAST as an adjunct in primary survey. A complete history, physical examination and imaging assessment of the chest, followed by chest X-ray which is the surrogate diagnostic standard in our setting was done. The findings for both investigations were recorded on the data tool. Two portable hand held ultrasound systems (Mindray DP-6600 FL, USA) were used in this study, one for each site. The device’s manufacturer has indicated eFAST as one of its uses [19] and has been validated in previous studies [20] in addition to being suitable in rural areas where there could be electricity blackouts [21].

### Ultrasound procedure

This procedure was performed in accordance with Taylor and O’Rourke [22]. The patient was asked to remove clothing and other objects such as jewelry that could interfere with the scan. Patient was positioned on examination bed either lying on back, or side or sitting up with arms raised with hands clasped around the neck depending on level of consciousness. Ultrasound gel was placed on area of chest to undergo examination. Using a transducer, ultrasound waves were sent from area being examined reflected off structures and analyzed by the ultrasound machine that created an image on the screen. The images generated were stored digitally. Patients were at times asked to cough or shift position or sniff for clarity of chest structures.

### Chest X-ray procedure

This was carried out in a radiology certified room with fixed X-ray machines. The patient was asked to undress, remove jewelry, stand (PA view) OR lie (lateral decubitus view) next to a cassette that recorded images for processing. For severely injured patients and suspected spinal injury patients, the X-ray tube and the image receptor were positioned, rather than the patient or the part to avoid the risk worsening the patient’s condition. Patient was instructed to roll shoulders forward, withhold breath, and stay still while image was being taken. The image was recorded on computer and printed on film for interpretation.

### Chest tube insertion procedure

This was done in the accidents and emergency department using the aseptic technique in the triangle of safety under local anesthesia according to the method described by Datta et al. [1]. All patients who had a hemothorax volume of greater than 300 mls had chest tube insertion since this volume is associated with a retained hemothorax if not drained [1]. Patients who had respiratory distress also underwent drainage irrespective of the volume quantified at sonography. Patients who had a volume less than 300 mls at sonography, but later deteriorated also underwent chest tube insertion.

### Quality control

The questionnaire was pretested at Ishaka Adventist Hospital to check whether it could extract the desired information on variables of interest and necessary changes were made. The investigator and trained research assistants (residents) collected the data. For every 5th patient, eFAST was assessed by a qualified radiologist. Where two radiologists did not agree on the radiological findings, the decision of an independent third radiologist was to be considered final. Data was checked for completeness at the end of definitive surgical intervention. The data was analyzed with the guidance of a biostatistician.

### Data analysis and presentation

Data was analyzed using SPSS version 22.0. Univariate analysis for continuous variables was summarized using mean and standard deviation, whereas proportions and percentages were computed for categorical variables and presented as frequency tables. The detection rates of haemothorax were computed individually for both CXR and eFAST with reference to the findings on chest tube drainage. The sensitivity, specificity, positive predictive value, negative predictive value and accuracy were calculated using the cross tabulation procedure and the corresponding chi-squire *P* values determined taking thoracostomy findings as the gold standard. *P* value of less or equal to 0.05 was considered significant for correlation between the detection rates of hemothorax by the investigation assessed and tube thoracostomy findings. The receiver operator characteristic curve (ROC) with the corresponding area under the curve (AUC) were used to compare the efficacy of the two investigations taking thoracostomy as the gold standard.

## Results

**Fig. 1:**
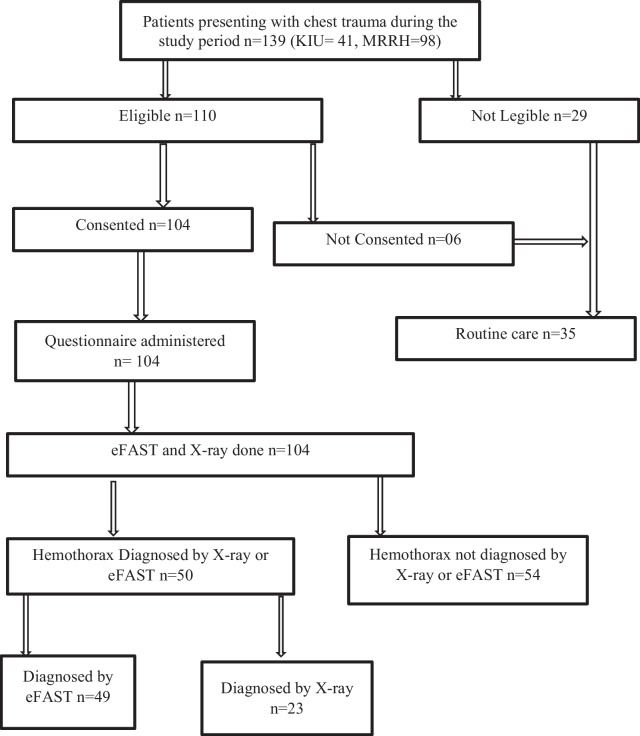
Showing the study procedure with the corresponding number of participants

During the study period, 139 patients presented to the study centres in total. Of these, only 110 were eligible for the study and of those eligible, only 104 consented to participate in the study. Figure 1 is a flow chart showing the study procedure with the corresponding number of participants.

### Characteristics of study participants

This study enrolled 104 study participants of whom majority were from Mbarara regional referral hospital (66.3%), Male (59.6%), from urban areas (51.9%) with mean age of 32 years. The commonest type of injury was blunt chest trauma accounting for 93.3% of the injuries and the commonest etiology was motor cycle crash (43.3%). The commonest associated injuries were found in the limbs (35.6%) and the commonest surgical intervention was tube thoracostomy done in 55 (52.9%) of the study participants. Table 1 shows the characteristics of study participants.

**Table 1:** Shows the characteristics of study participants

Characteristic	Frequency (n)	Percentage (%)
*Hospital*		
KIU-TH	35	33.7
MRRH	69	66.3
*Sex*		
Male	62	59.6
Female	42	40.4
*Education level*		
Not attended	27	26.0
Primary	24	23.1
Secondary	23	22.1
University	16	15.4
Other trainings	14	13.5
*Employment status*		
Employed	32	30.8
Un employed	72	69.2
*Occupation*		
Student	14	13.5
Civil servant	9	8.7
Casual labor	23	22.1
Private employee	26	25.0
Business man/woman	11	10.6
Farmer	21	20.2
*Residence*		
Rural	50	48.1
Urban	54	51.9
*Injury type*		
Penetrating	7	6.7
Blunt	97	93.3
*Etiology*		
Motor vehicle crash	22	21.2
Motor cycle crash	45	43.3
Falls	17	16.3
Sports injuries	6	5.8
Stab wounds	3	2.9
Others	11	10.6
*Associated injuries*		
No associated injury	21	20.2
Head	13	12.5
Spine	1	1.0
Neck	3	2.9
Abdominal	16	15.4
Pelvic	9	8.7
Limbs	37	35.6
Abdomen + limb	1	1.0
Spine + limb	1	1.0
Head + limb	1	1.0
Head + Abdomen	1	1.0
*Surgical intervention*		
No surgical intervention	41	39.4
Chest tube	52	50.0
STS	8	7.7
Chest tube + STS	3	2.9
Age (years)	Mean = 32.6, SD = 11.5	

*SD*, Standard deviation, *STS*, surgical toilet and suturing, *Min*, minimum, *Max*, Maximum

### Comparison of detection rates of haemothorax between eFAST and CXR in relation to tube thoracostomy findings

In this study, 55 (52.9% of the study participants) had thoracostomy done and of these, haemothorax was found in 51(49%). All study participants had both a chest X-ray and an eFAST done. eFAST found haemothorax in 47.1% of the study participants and X-ray in 22.1%. 48.1% of the participants were found to have a haemothorax by X-ray, eFAST or both. Table 2 Shows number of patients found with haemothorax by eFAST, X-ray and Thoracostomy.

**Table 2:** Shows number of patients found with haemothorax by eFAST, X-ray and Thoracostomy

Modality	Frequency	Percentage
*Thoracostomy*		
Hemothorax	51	49.0
No hemothorax	4	3.8
Thoracostomy not done	49	47.1
*eFAST*		
No hemothorax	55	52.9
Hemothorax	49	47.1
*X-ray*		
No hemothorax	81	77.9
Hemothorax	23	22.1
*eFAST and X-ray*		
No hemothorax	54	51.9
Hemothorax present	50	48.1

Taking thoracostomy findings as the gold standard, combining eFAST and X-ray had the highest sensitivity, Negative predictive value and accuracy, followed by eFAST then X-ray which had the lowest. All tests had specificity and positive predictive value of 100%. There was no significant relationship between the findings on X-Ray and thoracostomy according to the chi-squire test, but the relationship between the eFAST and thoracostomy findings was significant *P* < 0.001. Table 3 Shows comparison of detection rates of haemothorax between eFAST and CXR in relation to tube thoracostomy findings.

**Table 3:** Shows comparison of detection rates of haemothorax between eFAST and CXR in relation to tube thoracostomy findings

Modality	Sensitivity (%)	Specificity (%)	PPV (%)	NPV (%)	Accuracy (%)	*P* value
X-ray	45.1	100.0	100.0	12.5	49.1	0.131
eFAST	96.1	100.0	100.0	66.7	96.4	< 0.001
X-ray + eFAST	98.0	100.0	100.0	80.0	98.2	< 0.001

*PPV*, Positive predictive value, *NPV*, Negative predictive value, *P* value of chi squire test

**Fig. 2:**
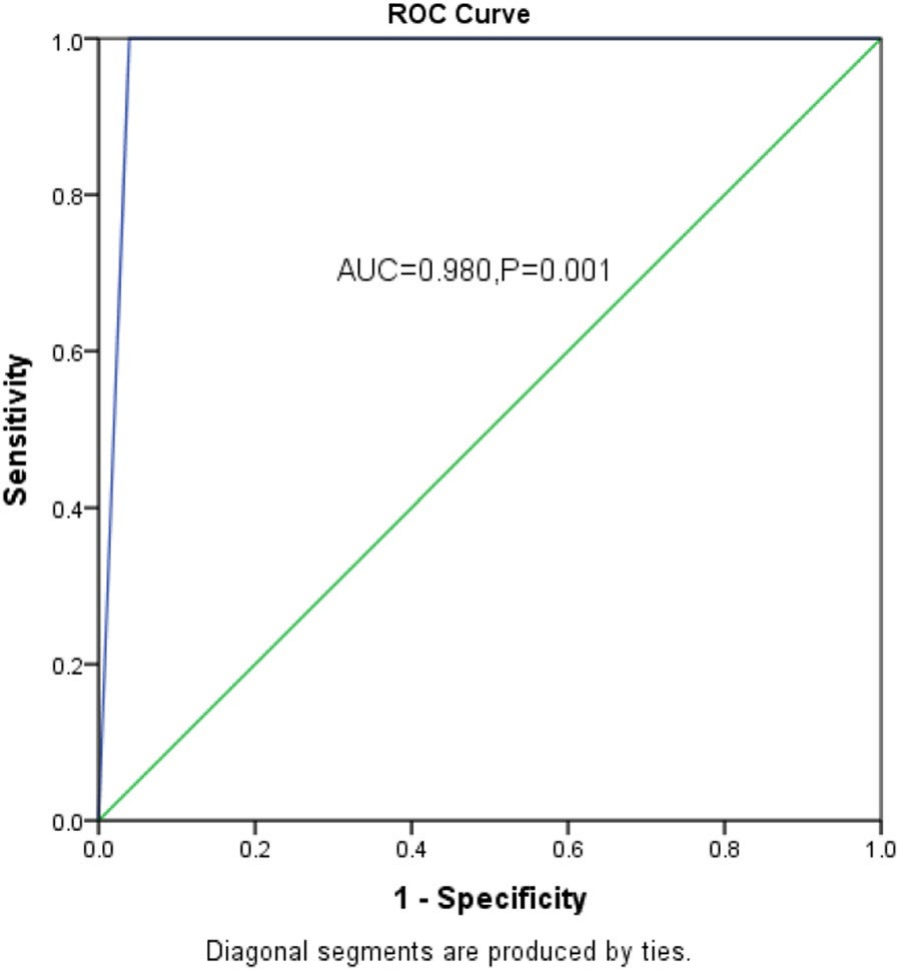
Shows the receiver operator characteristic curves for eFAST taking thoracostomy findings as the gold standard

**Fig. 3:**
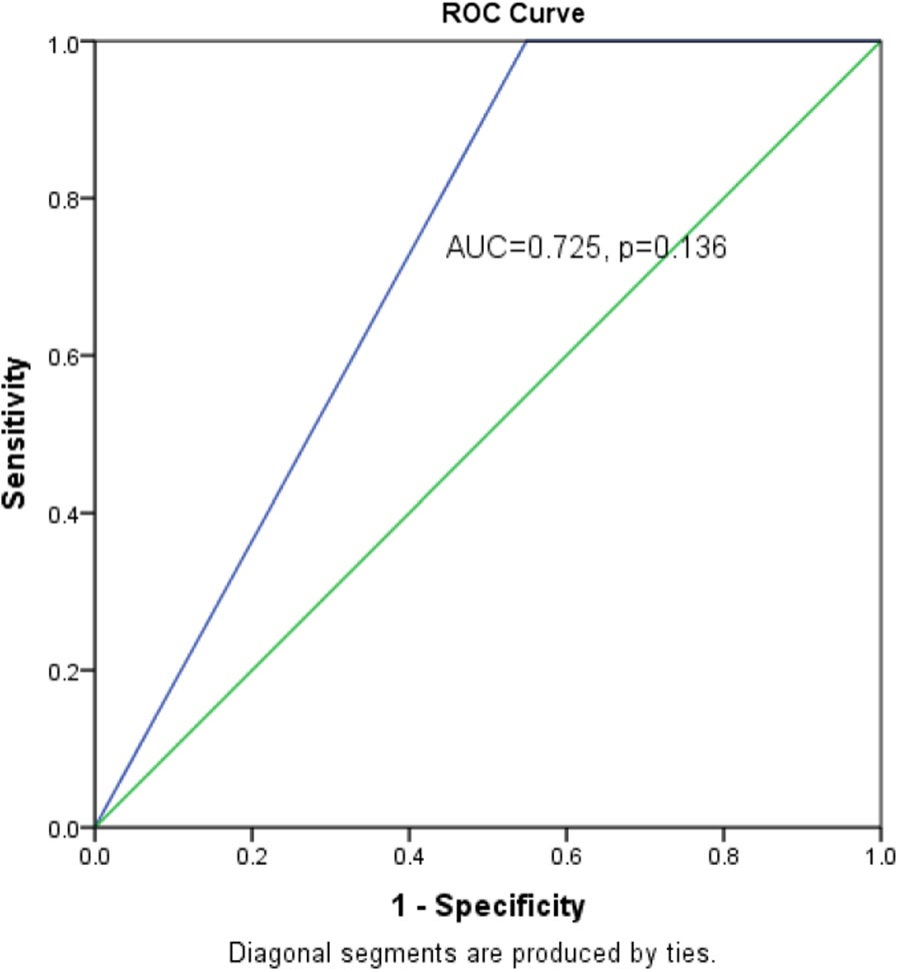
Shows the receiver operator characteristic curves for X-ray taking thoracostomy findings as the gold standard

The area under the curved for eFAST was much higher than that for X-ray (0.980, *P* = 0.001 vs. 0.725, *P* = 0.136). Figures 2 and 3 show the receiver operator characteristic curves for eFAST and X-ray respectively, taking thoracostomy findings as the gold standard.

The average volume of hemothorax not detected by X-ray but detected by sonography was 416.5 millilitres and the average volume of hemothorax detected by both sonography and chest X-ray was 769.8 millilitres. This difference was significant with a *P* value of < 0.001 using the independent samples t test. This big difference could have been because some patients could not sit upright and X-ray had to be done in supine position reducing detection rates further.

**Fig. 4:**
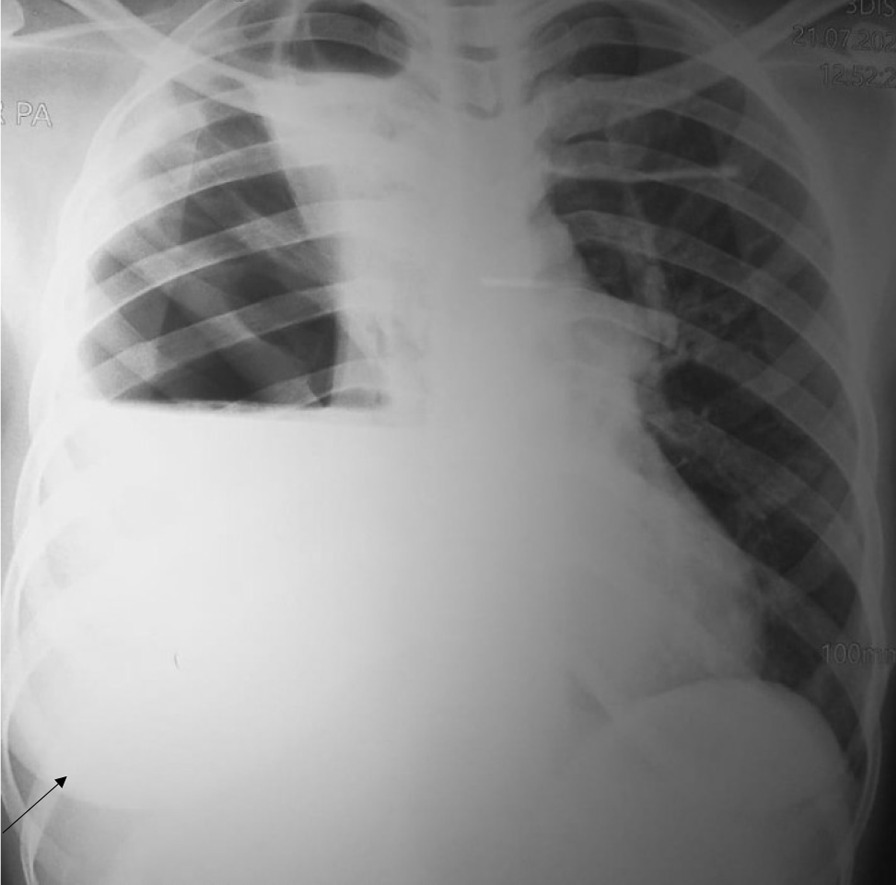
Shows a chest X-ray of right haemothorax in a participating 32 year old male

**Fig. 5:**
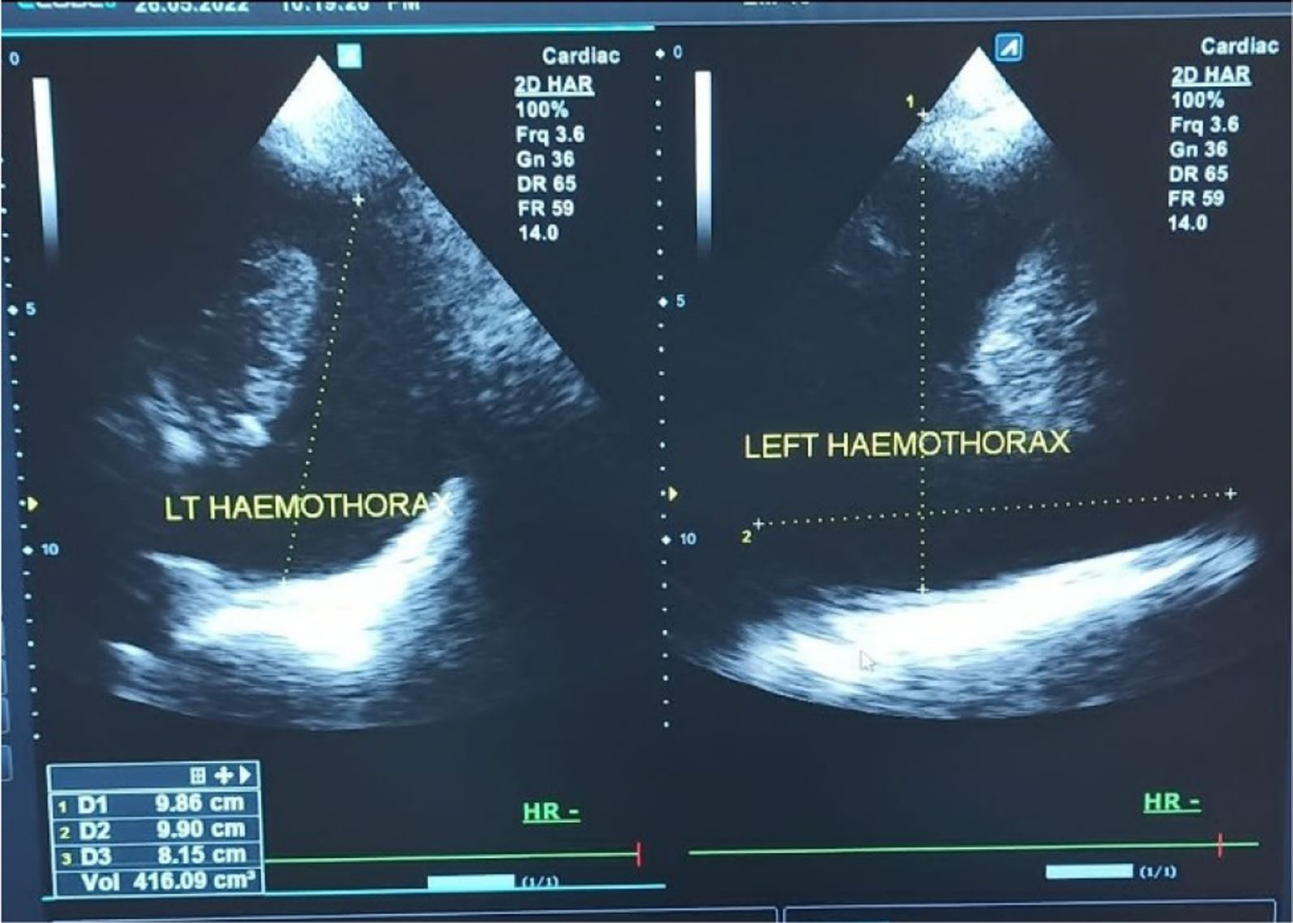
Shows a sonographic image of left haemothorax in a 30 year old male participant

Figure 4 shows a chest X-ray of right haemothorax in a participating 32 year old male and Fig. 5 shows a sonographic image of left haemothorax in a 30 year old male participant.

## Discussion

In this study, eFAST was more sensitive at detecting hemothorax than chest X-ray with sensitivity of 96.1% versus 45.1% respectively. The accuracy was also higher for eFAST (96.4% versus 49.1%) but the specificity was the same at 100.0%.

Our findings are comparable to the findings by Zieleskiewicz et al. [23] in France who also observed that eFAST was superior to X-ray with sensitivity of 48% versus 29%, and specificity of 100% for both tests. The sensitivities of ultrasonography and X-ray were much lower in the France study compared to our findings possibly because they used computed tomography as a gold standard yet we used thoracostomy findings as our gold standard.

The findings in our study are also comparable to the findings by Talari et al. [24] in Iran who reported that eFAST was superior to X-ray with sensitivity of 79% versus 36.9% and accuracy of 90.2% versus 71.1% and specificity of 99.1% for both eFAST and X-ray. However the values in our study are higher than those in the Iran study possibly because the Iran study used CT scan as the gold standard and not thoracostomy as was in our study.

The study findings by Attia and Gwely [25] in Egypt are also comparable to our findings where eFAST was reported superior to chest X-ray with sensitivity of 86.2% versus 58.6%, accuracy of 96.3% versus 89% and specificity of 100% for both investigations. Another study in Egypt that assessed the value of eFAST reported that eFAST was highly sensitive but more sensitive on the left side of the chest with sensitivity of 100% versus 93% on the right, accuracy of 97% versus 96% and specificity of 100% on both the left and right side of the chest, however the explanation or the theory behind the differences was not reported [26]. The sensitivity of chest X-ray was not reported in this study.

The low sensitivity of chest X-ray is because X-ray of chest in standing posture requires a collection of more than 400 ml of blood to obliterate the costophrenic angle while chest X-ray in supine position may not detect up to 1 L of blood as reported by Bhattacharyya and Brahma [27]. In this study, the patients who were fully conscious were asked to stand (PA view) or lie (lateral decubitus view) but in some patients especially those unconscious, the standing position could not be assumed which could have contributed to the low sensitivity of the chest X-ray. The high sensitivity of eFAST is because ultrasound can detect 100 ml of pleural fluid with 100% accuracy and also detect haemothorax as little as 20 ml according one review of literature by Zeiler et al. [1].

### Study limitations

CT scan was not done in this study yet it is the currently accepted gold standard for diagnosis of haemothorax, though only in stable patients. Therefore these results should be interpreted cautiously in the context of resource limited settings where routine access to chest CT scans cannot be guaranteed.

Also the principal investigator and assistants had inadequate experience in eFAST but this was mitigated by training before the study and having a qualified radiologist confirm the findings.

## Conclusion

This study revealed that eFAST was more sensitive at detecting haemothorax among chest trauma patients compared to chest X-ray. Portable hand held ultrasound systems have a big role to play in the diagnosis of hemothorax in stable and unstable patients.

### Recommendations

We recommend that all patients presenting with chest trauma should have an emergency eFAST for diagnosis of haemothorax preferably at all points of care especially with portable hand held ultrasound systems to minimize unnecessary exposure to radiations. Surgeons, Residents and Doctors involved in initial management of trauma patients should have skill and equipment (Portable bedside or point of care ultrasound systems) to adequately and promptly use eFAST to detect and manage hemothorax in trauma patients, in a timely and safe fashion.

## Data Availability

Data is available upon request. Requests should be sent to SMK Via doctormbaewood@gmail.com.

## Abbreviations

eFAST: Extended focused assessment with sonography for trauma
CXR: Chest X-ray
PPV: Positive predictive value
NPV: Negative predictive value
AUC: Area under the curve
ROC: Receiver operator characteristic curve
*P-*value: Probability value
